# Energy transfer from phycobilisomes to photosystem I at room temperature

**DOI:** 10.3389/fpls.2023.1300532

**Published:** 2024-01-08

**Authors:** Avratanu Biswas, Parveen Akhtar, Petar H. Lambrev, Ivo H.M. van Stokkum

**Affiliations:** ^1^ Department of Physics and Astronomy and LaserLaB, Faculty of Science, Vrije Universiteit Amsterdam, Amsterdam, Netherlands; ^2^ Insitute of Plant Biology, HUN-REN Biological Research Centre, Szeged, Hungary; ^3^ Doctoral School of Biology, University of Szeged, Szeged, Hungary

**Keywords:** photosystem I (PSI), global analysis (GA), target analysis, phycobilisomes (PBs), energy transfer (ET)

## Abstract

The phycobilisomes function as the primary light-harvesting antennae in cyanobacteria and red algae, effectively harvesting and transferring excitation energy to both photosystems. Here we investigate the direct energy transfer route from the phycobilisomes to photosystem I at room temperature in a mutant of the cyanobacterium *Synechocystis* sp. PCC 6803 that lacks photosystem II. The excitation dynamics are studied by picosecond time-resolved fluorescence measurements in combination with global and target analysis. Global analysis revealed several fast equilibration time scales and a decay of the equilibrated system with a time constant of ≈220 ps. From simultaneous target analysis of measurements with two different excitations of 400 nm (chlorophyll a) and 580 nm (phycobilisomes) a transfer rate of 42 ns^-1^ from the terminal emitter of the phycobilisome to photosystem I was estimated.

## Introduction

**Figure 1:**
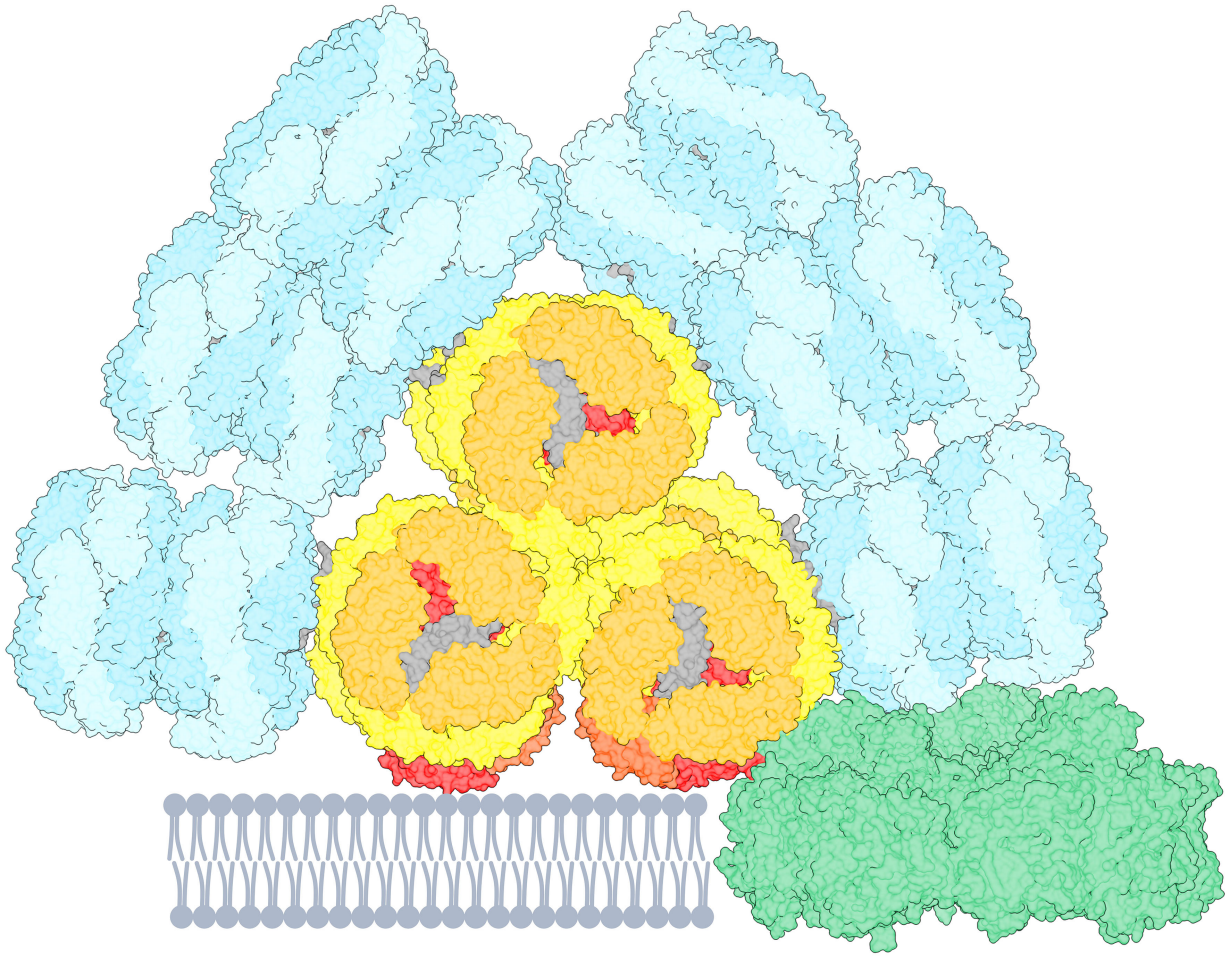
Cartoon representation of a PB-PSI complex with PSI trimer (green, PDB 5oy0, Malavath et al., 2018). The PB structure (PDB 7ext, Zheng et al., 2021) consists of rods (cyan) containing PC640 and PC650. The three-cylindrical APC core (yellow–orange) contains APC660 and the red-shifted emitters APC680, presumably located in the subunits ApcD, ApcE, ApcF (red and orange-red). The figure is created using UCSF ChimeraX (Goddard et al., 2018). The pigment stoichiometry is detailed in **Supplementary Figure 1**.

The primary light-harvesting antennae in cyanobacteria, rhodophytes, and glaucophytes are known as phycobilisomes (PBs). These supramolecular water-soluble pigment-protein complexes are located on the stromal side of the thylakoid membranes (Gantt and Conti, 1966; MacColl, 1998). Recent cryo-EM structural data (Domínguez-Martín et al., 2022) propose a 6.2 MDa PB from *Synechocystis* sp. PCC 6803 (hereafter denoted as *Synechocystis*) with an overall resolution of 2.1-3.5 Å. The PB are formed by phycobiliproteins, consisting of two polypeptide subunits designated as α and β, often forming a heterodimer (αβ), each covalently binding open chain tetrapyrrole chromophores (phycobilin) and colorless linker proteins (Ducret et al., 1994; MacColl, 1998; Adir, 2005; Bar-Eyal et al., 2018; Adir et al., 2020). The αβ heterodimers self-assemble into either disc-like trimers (αβ_3_) or further aggregate into hexamers [(αβ_3_)]_2_, serving as the basic building blocks of PBs. The PB structure consists mainly of two distinct substructural and functional domains: the allophycocyanin (APC) core, located adjacent to the membrane, and peripherally attached phycocyanin (PC) rods. The core is formed by four trimers of APC heterodimers (absorption λ_max_ ≈ 650 nm, emission λ_max_ ≈ 660 nm) stacked up together forming cylinders. The rods, composed of two or three PC (occasionally phycoerythrin) hexamers (absorption λ_max_ = 620 nm, emission λ_max_ = 645 nm) connected by linker proteins extend outwards from the core forming a hemidiscoid fan-like structure. The *Synechocystis* PB core comprises of three cylinders: top and two basal cylinders, with six rods attached (**Figure 1**, **Supplementary Figure 1**). The organization of the rods can vary based on the growth conditions (Grossman et al., 1993).

The PBs constitute an energy funnel, where the distal rods with PC pigments absorb light at the shortest wavelengths and the APC core absorbs at the longest wavelengths and lowest photon energy. Moreover, the core consists of modified APC proteins, ApcD, ApcE and ApcF, which possess a red-shifted spectrum (Absorption λ_max_ = 670 nm, Emission λ_max_ = 680 nm) compared to the bulk APC. The low-energy APC pigments act as the terminal emitters facilitating excitation energy transfer (EET) to chlorophylls (Chls) in the photosystems (Ashby and Mullineaux, 1999; Tian et al., 2011; Liu et al., 2013). PBs are associated to photosystem II (PSII) directly (Arteni et al., 2009; Chang et al., 2015; Rast et al., 2019) and serve as the external light-harvesting antenna of PSII albeit they can also interact and transfer energy to photosystem I (PSI) (Mullineaux and Holzwarth, 1993; Liu et al., 2013; Noji et al., 2021). In non-optimal light conditions, the excitation energy redistribution between PSII and PSI is regulated by a mechanism called state transitions (Bruce et al., 1989; Chukhutsina et al., 2015; Bhatti et al., 2020). The energy harvested by PBs can also be transferred to PSI either directly via energetic coupling between PSI and PBs (Su et al., 1992; Mullineaux, 1994; Liu et al., 2013), or via an indirect route involving the ‘spillover’ of energy from PSII to PSI (McConnell et al., 2002; Ueno et al., 2016; Ueno et al., 2017). **Figure 1** is inspired by the PB-PSIPSII megacomplex discovered by (Liu et al., 2013).

Steady-state and time-resolved fluorescence spectroscopy techniques have been performed to assess the energetic coupling between PB and PSI in cyanobacteria. Mullineaux (Mullineaux, 1994) reported that, at 77K, PSII-deficient *Synechocystis* cells exhibit a quantum efficiency of around 80% for PBS-to-PSI EET, contrasting with the 40% observed in wild-type (WT) cells. Conversely, room temperature picosecond studies, combined with functional kinetic modeling of experimental data, estimated a 50% EET to PSI within a PB-PSII-PSI megacomplex (Tian et al., 2011; Biswas et al., 2020). In *Synechococcus elongatus* sp. PCC 7002, the deletion of ApcD reduces EET to PSII, directly impacting cyanobacterial growth rates (Dong and Zhao, 2008). Although there is a wealth of experimental data and various models available, there is no general consensus regarding the kinetics and rate limitations of excitation migration within PBs and between PBs and the photosystems.

The organization and function of the cyanobacterial light-harvesting complexes and reaction centers can be tested by combining optical spectroscopy with molecular genetics approaches. A detailed functional compartmental model of the PBs of *Synechocystis* WT and mutants lacking entirely the rods (van Stokkum et al., 2018) revealed various EET time constants between the core cylinders (115-145 ps) and between the rods and the core (68-115 ps). Similarly, an inverted kinetic process of EET was observed in a PSI-deficient mutant of *Synechocystis*, indicating accelerated EET from the terminal emitter of the PBS to PSII (with rate (20 ps)^-1^) compared to intraphycobilisome EET rates (Acuña et al., 2018). It has been shown that the *Synechocystis* mutants lacking PSII retain well-assembled PBs without having an effect on the excitation dynamics (Bittersmann and Vermaas, 1991). However, these mutants exhibited higher PBs fluorescence intensity compared to WT *Synechocystis* but less then isolated PBs – indicating EET from PBs to PSI.

In the present study, we investigated EET at room temperature from PBs to PSI in a mutant of the cyanobacterium *Synechocystis* which lacks PSII. We performed time-resolved fluorescence measurements on whole cells and on isolated PSI and PB under the same conditions. By simultaneously modeling both the isolated and *in vivo* systems, we successfully estimated the path and the rate of EET from PB to PSI in the PSI-PB megacomplex (**Figure 1**, **Supplementary Figure 1**).

## Materials and methods

### Culture conditions

Cyanobacterial cultures of *Synechocystis* sp. PCC 6803 wild type (WT) and a PSII-deficient mutant, referred to as ΔPSII, were used in this study. The mutant strain, provided by Wim Vermaas, was generated by deleting the psbA, psbC, and psbD genes, leading to the absence of crucial PSII proteins, including D1, D2, CP43, and CP47 (Yu and Vermaas, 1990). The cells were cultivated in BG11 medium supplemented with 10 mM glucose, 40 µg/ml spectinomycin, and 8 µg/ml chloramphenicol. The cultures were incubated on a rotary shaker at 100 rpm and 30°C, with continuous white light exposure at an approximate intensity of 35 μmol photons m^−2^ s^−1^.

### Sample preparations

The preparation of PB was carried out following the protocol described by Garnier et al. (1994) with some modifications. Cells were pelleted by centrifugation at 7,000 g at room-temperature and washed twice with phosphate buffer (0.75 M phosphate buffer, 1 mM benzamidine hydrochloride hydrate, 1 mM EDTA, pH 7.0, and 1 mM phenylmethylsulfonyl fluoride). The pellet was treated with 0.2% (w/v) of lysozyme and incubated for 1 h in the dark at 37°C with continuous shaking at 200 rpm. After incubation, cells were pelleted down by centrifugation at 6,000 g for 7 min at 14°C and washed twice with phosphate buffer to remove the remaining lysozyme. The cells were then broken with glass beads (≤ 106 μm diameter) using a homogenizer (Precellys Evolution) equipped with a dry-ice-cooling compartment. The remaining glass beads were removed by centrifugation at 3,000 g for 5 min at 14°C. The supernatant was incubated with 3% (v/v) Triton X-100 for 30 min at room temperature in the dark and centrifuged at 21,000 g for 30 min to remove the unsolubilized material. The upper greenish layer was discarded and the supernatant loaded onto a sucrose density step gradient (0.25, 0.5, 0.75, and 1 M) and centrifuged for 16 h at 26,000 g at 14°C for further purification. The gradient fraction containing PBs was collected and characterized by steady-state spectroscopy for further usage.

Thylakoid membranes and PSI complexes were prepared as described by Akhtar et al. (2021). Briefly, the membranes were solubilized by incubating with 2% n-dodecyl β-D-maltoside (β-DDM) at 4°C for 30 minutes. The suspension was centrifuged at 10,000 g for 15 minutes to remove unsolubilized material. The supernatant was then loaded on a step sucrose density gradient (six steps, 0.2–0.9 M) containing 20 mM HEPES (pH 7) and 0.05% β-DDM, and centrifuged at 220,000g for 17-18 hours at 4°C. Fractions of the gradient containing PSI trimers were collected by Hamilton syringe. The samples were washed in a medium containing 0.03% β-DDM, concentrated using Amicon Ultra filters (Millipore), and stored at -80°C until further use.

### Steady-state absorption and fluorescence spectroscopy

The absorption spectra at room temperature were recorded using a Thermo Evolution 500 dual-beam spectrophotometer in the range of 350-750 nm. The measurements were carried out with a standard glass cell having an optical path length of 1 cm and a spectral bandwidth of 1 nm. The fluorescence emission spectra in the visible range were measured at the room-temperature using FP-8500 (Jasco, Japan) spectrofluorometer. The samples were diluted to an absorbance of 0.1 per cm at the red maximum. The emission spectra from 600-780 nm were recorded with excitation wavelengths of 400 and 580 nm, and an excitation/emission bandwidth of 2.5 nm. The measurements were carried out with 1 nm increment and 1-s integration time. The spectra were corrected for the spectral sensitivity of the instrument using a calibrated light source (ESC-842, Jasco) as a reference.

### Time-resolved fluorescence spectroscopy

Picosecond time-resolved measurements were carried out using a synchroscan streak-camera setup described elsewhere (van Stokkum et al., 2008; Acuña et al., 2018; Biswas et al., 2020). Briefly, the femtosecond laser source, Coherent Vitesse Duo, pumped the regenerative Coherent RegA 900, and the output from the RegA further fed into the optical parametric amplifier Coherent OPA 9400, which had an output wavelength range from 470 to 770 nm. Vertically polarized emission was collected at a right angle to the incident beam by a spectrograph (Chromex 250IS, 50 grooves/mm, blaze wavelength 600 nm, and spectral width of each image 260 nm). The central wavelength was set at 682 nm for all experiments. The light was focused on the input slit of 80 μm and further onto the photocathode of the streak camera (Hamamatsu C5680). Laser pulses with repetition rate of 250 kHz and centered at a wavelength of 532, 540 or 580 nm, selected using the OPA and an additional bandpass filter, or 400 nm, the frequency-doubled output of the RegA, were used to preferentially excite the PBs or Chls, respectively. The laser power was 15 μW for PSI, 30 μW for PB and intact cells. The laser spot size was ≈50 μm diameter in all excitations. All samples were diluted to an optical density of 0.3 at 680 nm and magnetically stirred at a very low speed to avoid any reabsorption or overexcitations.

Fluorescence was recorded from 590 to 860 nm and 0 to 500 ps. The full width at half maximum (FWHM) of the instrument response function (IRF) was ≈8 ps. A sequence of 360 images was collected - with each image resulting from a scan of 10 s. Before the analysis, image sequences were averaged and corrected for background, signal jitter and camera shading using HPD-TA software (Hamamatsu). The corrected datasets were sliced into traces of 3 nm width using Glotaran 1.5.1 (Snellenburg et al., 2012). The datasets were further analyzed using PyGlotaran (van Stokkum et al., 2023c).

### Data analysis

The global and target analysis of time-resolved spectroscopy measurements aims to solve an inverse problem of decomposing time-resolved spectra into the superposition of contributions from different components **Formula (1)**


(1) $$\begin{array}{c} \Psi \left(\text{t},\text{λ}\right)=\sum_{i=1}^{n}C_{i}\left(\text{t},\text{θ}\right)E_{i}\left(\text{λ}\right) \end{array}$$

where *n* is the number of components in the system, $C_{i}\left(\text{t},\text{θ}\right)$ is the *i*-th time dependent concentration profile described by nonlinear parameters *θ*, and $E_{i}\left(\lambda \right)$ is the *i*-th spectrum. This problem is solved by estimation of the unknown parameters *θ* using the Variable Projection algorithm (Golub and LeVeque, 1979; Mullen and van Stokkum, 2009).

#### Global analysis

In global analysis, the concentration profile *C_i_* is modeled as the convolution of an exponential decay and IRF(t) . The associated spectra are called Decay Associated Spectra (DAS) in this case, then the **Formula (1)** is given by


$$\text{Ψ}\left(\text{t},\text{λ}\right)=\sum_{i=1}^{n}e^{-k_{i}t}\;*\text{IRF}\left(t\right)\cdot {\text{DAS}}_{i}\left(\lambda \right)$$

where * indicates the convolution. $k_{i}$ are the decay rates, which are the inverse of the fluorescence lifetime ( $\tau_{i}$ ). Negative and positive amplitudes of the DAS correspond to rise and decay of emission, respectively, and indicate EET from the donor to the acceptor (Holzwarth, 1996).

#### Target analysis

In the case of target analysis of time-resolved emission spectra, the concentration profiles $C\left(t\right)={\left[C_{1}\left(t\right),C_{2}\left(t\right),\ldots ,C_{n}\left(t\right)\right]}^{T}$ are the solutions of parametric first-order ordinary differential equations


$$\frac{\text{d}}{\text{d}t}C\left(t\right)=KC\left(t\right)+\text{IRF}\left(t\right)j$$

with the initial condition


C(t=−∞)=0

The non-zero off-diagonal element $k_{pq}$ in the coefficient matrix ***K***, is the EET rate from component *q* to component *p*, and diagonal element $k_{pp}$ represents the decay of component *p* caused by fluorescence or trapping. The initial condition $j={\left[j_{1},\;j_{2},\;\ldots ,j_{n}\right]}^{T}$ represents the initial excitation of each component.

In target analysis, the spectra $E_{i}$ , called species-associated spectra (SAS), reflect the emission spectrum of each compartment (pigment pool). When a sequential irreversible kinetic scheme with increasing lifetimes is used, the SAS are called evolution-associated spectra (EAS). Because the EAS and SAS represent emission spectra, we employ a non-negative least-squares constraint in the optimization process (Mullen et al., 2007; Mullen and van Stokkum, 2009) for these conditionally linear parameters. Additionally, to estimate the equilibria, equality constraints on the SAS integrated area are imposed (Snellenburg et al., 2013).

#### Residual analysis

Residual analysis involves applying singular value decomposition to the matrix of residuals (***R***) obtained from the fitted data. The expression for the residual matrix (*R*) is given by,


$$R\left(t,\lambda \right)=\sum_{i=1}^{m}lsv_{i}\left(t\right)\cdot s_{i}\cdot rsv_{i}\left(\lambda \right)$$

The left singular vectors ( lsv ) capture time-dependent information, while the right singular vectors (*rsv*) contain information specific to wavelength *λ*, revealing patterns and trends specifically related to different spectral components. The singular values $s_{i}$ associated with each singular vector quantify the significance of their respective contributions to the overall matrix approximation. Notably, the *lsv* and *rsv* corresponding to the largest singular value show the main trends of the residual matrix in the time or wavelength dimension.

#### Simultaneous target analysis

Simultaneous target analysis was conducted on selected datasets from each type of experiment. The simultaneous target analysis has the advantage of combining the information accessible by different experiments (different excitation conditions, etc.) and in this way extracting a more detailed picture of the excitation dynamics. The simultaneous target model is represented as:


$$\left[\begin{array}{c} {\text{Ψ}}_{1}\left(t,\lambda \right)\\ {\text{Ψ}}_{2}\left(t,\lambda \right)\\ \vdots \\ {\text{Ψ}}_{m}\left(t,\lambda \right) \end{array}\right]=\left[\begin{array}{c} C_{1}\left(t\right)\\ {\text{α}}_{2}C_{2}\left(t\right)\\ \vdots \\ {\text{α}}_{m}C_{m}\left(t\right) \end{array}\right]\cdot \text{SAS}\left(\lambda \right)$$

Here, *m* represents the number of datasets and **C** *_i_* denotes the concentration matrix of experiment *i* as explained in the previous section. The key constraint in simultaneous analysis is that the SAS matrix is shared among all datasets, i.e. the compartments in all datasets represent the same species across experimental conditions. A scaling factor *α* is introduced for each extra dataset to account for relative size variations in measurements. The simultaneous target analysis helps in identifying different plausible species present and improves the precision of the estimated parameters. In the context of our study, since two different excitation wavelengths result in varying initial concentrations of the compartments, the spectrally identical species can be better resolved through such an analysis.

## Results and discussion

### Fluorescence study

**Figure 2:**
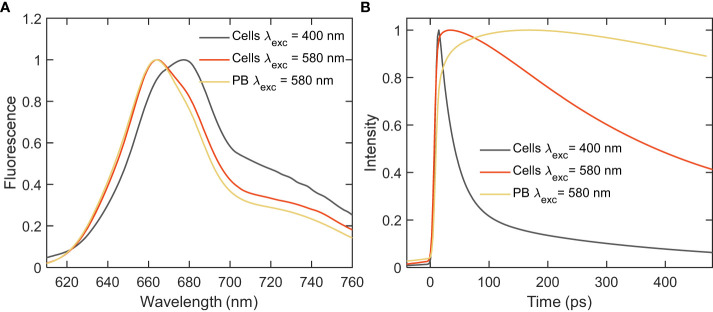
Fluorescence emission spectra of intact cells and isolated PBs of *Synechocystis* ΔPSII recorded at room-temperature with Chl (400 nm) and PB (580 nm) excitations. **(A)** Steady-state emission spectra normalized at the emission maximum; **(B)** Integrated decay traces obtained from the time-resolved emission spectra between wavelengths of 680-720 nm.

Steady-state fluorescence emission spectra of whole *Synechocystis* cells recorded at room temperature with excitation either at 400 or 580 nm - exciting predominantly Chls or PBs, respectively, are shown in **Figure 2**. For comparison, the spectrum of isolated PBs is also plotted. Both excitations resulted in broad Chl fluorescence emission in the red region with emission maximum position changing with the excitation wavelength. The emission spectrum with Chl excitation shows a broad peak at 680 nm and a shoulder at 665 nm, originating from a mixture of PC and APC. The emission spectrum recorded with PB excitation shows a peak at 665 nm and a shoulder at 680 nm – reflecting combined emission from Chls in PSI and the terminal emitters of PB. The strong emission at 660 nm observed even with predominant excitation of Chl suggests that not all energy absorbed by PBs is transferred to PSI.

To study the EET process within the mutant cells picosecond time-resolved fluorescence was measured with a synchroscan streak camera system. Although little to no significant direct evidence of EET was obtained from the steady-state fluorescence measurements, the decay kinetics integrated over the wavelength range of 680-720 nm (**Figure 2B**), revealed a distinct decay of excitations upon PB excitation - much faster than the decay of isolated PB (lifetime ≈1.5 ns), which provides first evidence that at least part of the energy absorbed by PB is transferred to PSI.

### Global analysis of the time-resolved fluorescence

**Figure 3:**
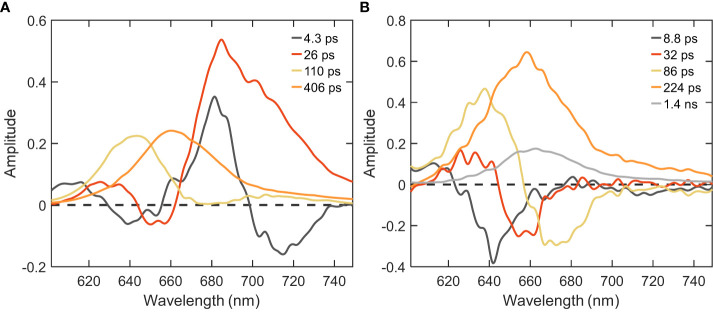
DAS of *Synechocystis* ΔPSII cells estimated from global analysis of time-resolved fluorescence decays measured at RT. **(A)** λ_exc_=400 nm; **(B)** λ_exc_=580 nm.

**Figure 4:**
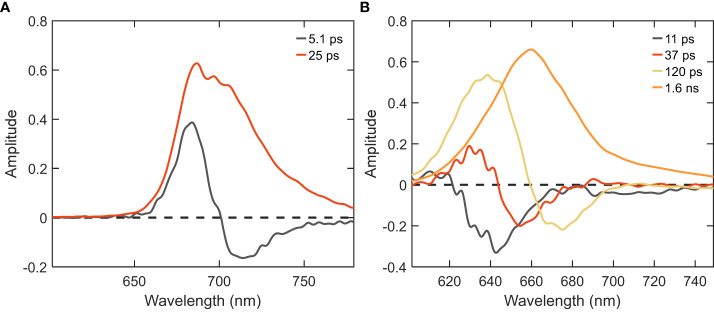
DAS of PSI and PB samples isolated from *Synechocystis* WT cells obtained from global analysis of time-resolved fluorescence decays measured at RT. **(A)** PSI excited with λ_exc_=400 nm; **(B)** PB excited with λ_exc_=580 nm.

Further, to disentangle the spectral and kinetic properties, we performed global analysis of the time-resolved fluorescence data. Integrated decay traces obtained from the time-resolved emission spectra between wavelengths of 680-720 nm clearly demonstrated the faster decay in intact cells compared to isolated PB (**Figure 2B**). The fluorescence kinetics recorded with 400 nm excitation can be described with four decay lifetimes (**Figure 3A**). The excitation dynamics are dominated by the EET and trapping in PSI. The fastest decay component with a lifetime of 4.3 ps shows both EET within the PB - a negative peak in the wavelength region below 660 nm - and EET from the Bulk to Red Chl pools of PSI (*λ*_max_ ≈680 and ≈720 nm). The 26 ps DAS with major positive amplitude belongs almost exclusively to excitation trapping by PSI. These two components have been well resolved in previous studies (Chukhutsina et al., 2013; Tian et al., 2013) and are in good agreement with the rapid EET processes observed within isolated PSI and *in vivo* WT cells (shown in the supplementary results having two major lifetimes of 5.1 and 25 ps, cf. **Figure 4**, **Supplementary Figure 2**). The two slower-decaying components with the lifetimes of 110 ps and 406 ps can be attributed to the decay of PB fluorescence, which can be better understood via the global analysis of the 580 nm excitation data.

With the 580 nm excitation, we primarily probe the EET within the PB antenna and from PB to the photosystems present in the cells (**Figure 3**). At least five components were necessary to describe the data. The absence of PSII, typically regarded as the natural energy acceptor for PB-harvested energy, results in minimal alterations observed in the PB excitation dynamics when comparing it to the WT (**Supplementary Figure 2**) or isolated PB (**Figure 4**). The first three components having positive peaks at shorter wavelength and negative peaks at longer wavelength can be attributed to EET and the last two components are associated with fluorescence decay. The two shortest lifetimes (≈9 and 32 ps) correspond to the EET between the different pools of PC rods. The third component with the 86 ps lifetime represents EET from the PC rods to the APC cores of the PB. The majority of APC excitations decay with a lifetime of ≈200 ps – substantially shorter compared to the 1.4 ns lifetime – indicating EET to the photosystems (cf. the orange and grey DAS in **Figure 3B**, **Supplementary Figure 2B**). The spectral shape of this component differs between WT and the mutant - a well-defined shoulder is visible at 680 nm in the WT due to the presence of PSII, which is lacking in the mutant. Despite the absence of PSII, the similar lifetimes in both cell types suggest the existence of an acceptor that mitigates the slow EET from the PB to the Chls of PSI in the mutant. The long-lived component of ≈1.4 ns can be assigned to the fraction of uncoupled PB (cf. the small grey DAS in **Figure 3B**). This component has a larger amplitude compared to WT (**Supplementary Figure 2**). From the comparison of the amplitudes of the 224 ps and 1.4 ns DAS, we can conclude that the majority of the energy harvested by PBs is transferred to PSI in the ΔPSII mutant.

### Simultaneous target analysis of the time-resolved fluorescence data

**Figure 5:**
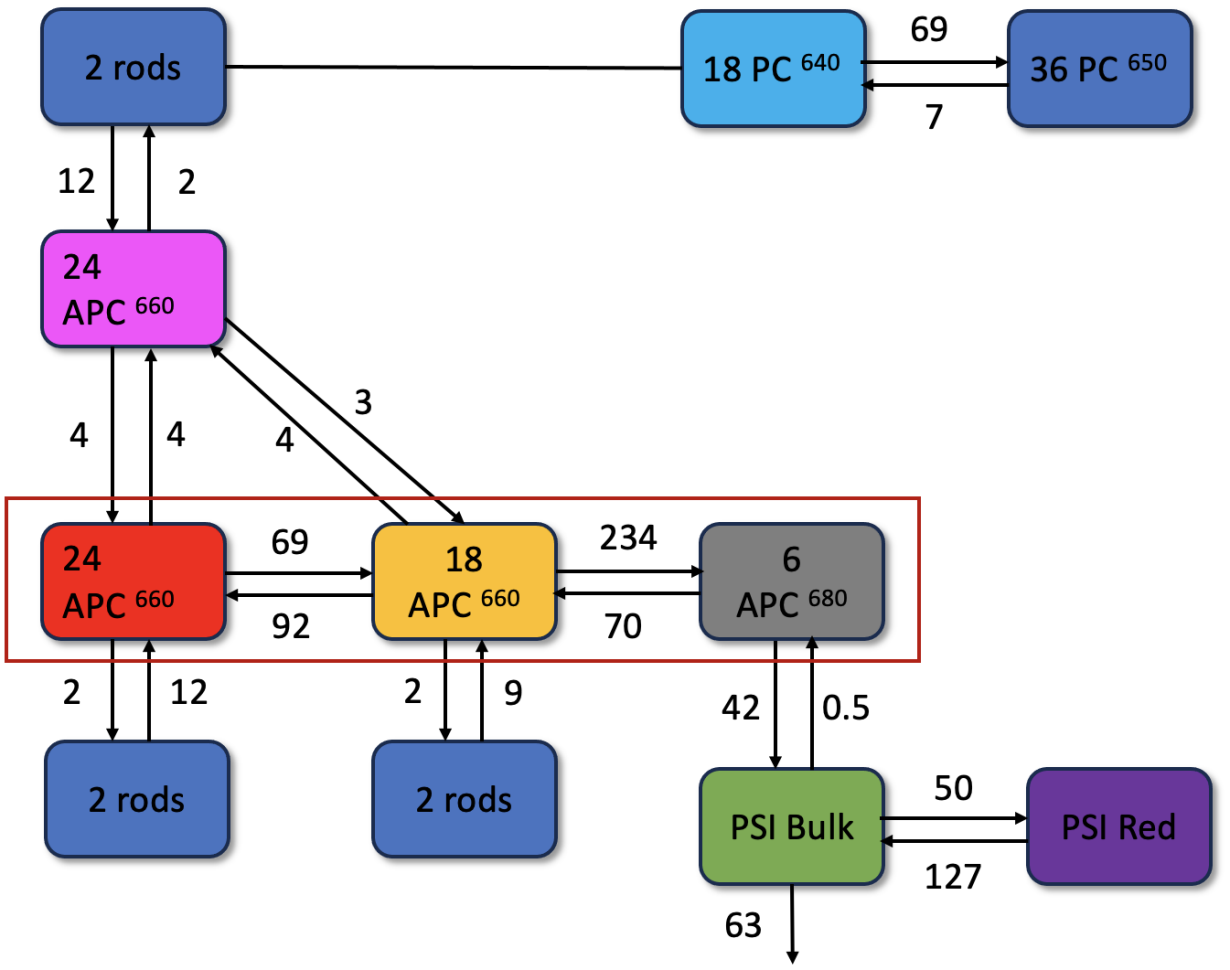
Functional compartmental model of *Synechocystis* ΔPSII cells, representing a minimal kinetic model of PB-PSI at room-temperature with microscopic rate constants in ns^-1^, with a zoom out of a rod consisting of three lumped hexamers in the upper right. The rods contain PC640 (cyan) and PC650 (blue). The magenta APC660 compartment represents the top cylinder. The red rectangle indicates the two basal cylinders, with APC660 (orange) and APC680 (black) in four disks, and APC660 (red) in four other disks. The PSI complex consists of bulk (green) and red (purple) Chl a compartments and is connected to APC680.

Based on the comprehensive global analysis of the *in vivo* fluorescence kinetics of cells with selective excitations and *a priori* knowledge from the literature (Tian et al., 2011; van Stokkum et al., 2018), we compartmentalize the whole cells into biophysically relevant units, which we refer to as megacomplexes. The ΔPSII mutant cells contain a mixture of PB-PSI complexes (**Figure 5**), non-transferring PB and Free rods. The model of the PB dynamics is based upon the structure (**Supplementary Figure 1**) and distinguishes six distinct functional compartments (**Figure 5**). The PB core exhibits a configuration of three functional APC660 (red and orange) compartments alongside one APC680 compartment (grey). Hexameric rods extending from the core, are comprised of two functional compartments, PC640 (cyan) and PC650 (blue). The kinetic model accounts for the presence of four rods connected to the basal cylinders and two rods connected to the top cylinder. Altogether, the PB model consists of ten functional compartments. The coupling of the PB structure with PSI is facilitated by core-membrane linker proteins, enabling efficient EET. Therefore, in our model, we established a connection between PB and PSI (PB-PSI megacomplex) to investigate direct evidence of EET. We employ a highly simplified model of PSI that consists of two functional compartments, namely the Bulk Chl a, including the reaction center (green), and Red Chl a (purple) compartments. With these data it was not possible to distinguish the reaction center compartment from the bulk antenna (van Stokkum et al., 2023b), and an effective rate constant of trapping by the combined antenna-reaction center compartment of 63 ns^-1^ was estimated, which agrees with previous studies (Chukhutsina et al., 2013; Tian et al., 2013). In agreement with these previous studies, the Gibbs free energy of the PSI Red Chl a compartment is higher than that of the PSI Bulk Chl a compartment because the entropy difference is larger than the enthalpy difference. Quantitatively, the enthalpy difference corresponds to ≈600 cm^-1^ or 2.95 k_B_T. The Gibbs free energy difference equals ln(127/50)=0.93 k_B_T. The entropy difference then corresponds to 3.88 k_B_T. The ratio of pigments in the PSI Bulk Chl a and Red Chl a compartments thus equals exp(3.88)=48, i.e. 96 and 2 pigments in the PSI Bulk Chl a (plus RC) and Red Chl a compartments.

The estimated amounts of non-transferring PB and Free rods megacomplex present with the different excitations are collated in **Table 1**. Simultaneous target analysis was conducted on the measured fluorescence kinetics of ΔPSII mutant cells with *λ_exc_* 580 or 400 nm and PB with *λ_exc_* 580 nm (**Supplementary Figure 3**). The selective excitation of PB or PSI with *λ_exc_* 580 or 400 nm helps to resolve the intricate dynamics of the PB-PSI complex.

**Table 1:** Estimated fractions of the three different complexes in ΔPSII.

Complex \ λ_exc_	400 nm	580 nm	540 nm	532 nm
*PB-PSI*	58.6%	54.6%	63.1%	60.5%
*PB*	40.7%	41.4%	31.9%	33.9%
*rod*	0.7%	4.0%	4.9%	5.7%

An alternative EET pathway was tested, as suggested in Figure 3E in (Liu et al., 2013), in a target analysis based upon a kinetic scheme with EET to PSI from the PC650 (of the bottom cylinders) instead of the APC680. The overall rms error of the fit increased from 0.061 to 0.102 (**Supplementary Figure 4**). The estimated EET rate from the PC650 (of the bottom cylinders) to PSI was 7.5 ns^-1^. The misfit was most clearly visible in the traces from 650 to 750 nm of 580 nm excited ΔPSII (orange in **Supplementary Figure 4**). Because of this misfit we must reject this alternative kinetic scheme with EET to PSI from the PC650 (of the bottom cylinders).

**Figure 6:**
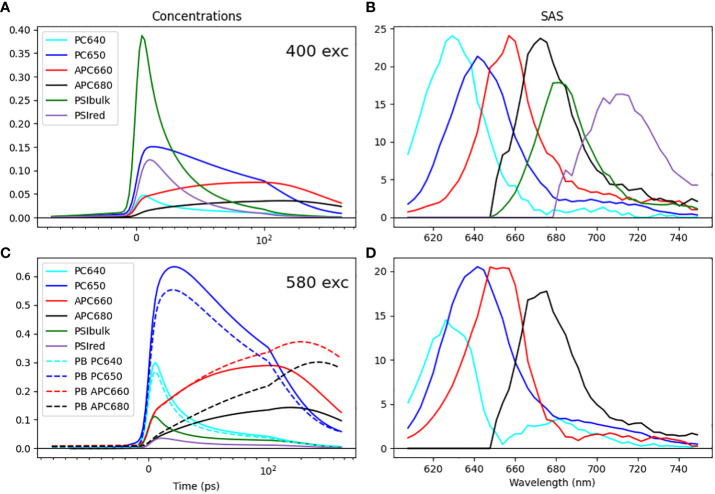
Simultaneous target analysis results of *Synechocystis* ΔPSII cells **(A–C)** and the PB complex **(C, D)** at room-temperature. Total concentrations estimated after 400 **(A)** or 580 **(C)** nm exc. SAS **(B, D)**. Key: PC640 (cyan), PC650 (blue), APC660 (red), APC680 (black), PSI Bulk Chl a (dark green), and Red Chl a (purple). Line type key in C: ΔPSII cells (solid), PB complex (dashed). Note that the time axis in A, C is linear until 100 ps and logarithmic thereafter.

Through simultaneous target analysis, the individual chromophore groups present within the *Synechocystis* ΔPSII cells could be resolved on the basis of their SAS (**Figure 6**). We found that the *in vitro* SAS of the isolated PB slightly differed from their *in vivo* counterparts (cf. panels D and B of **Figure 6**). We employed guidance spectra (van Stokkum et al., 2022; van Stokkum et al., 2023c) to minimize these differences. The ten PB compartments contained four different SAS: PC640, PC650, APC660 and APC680. Together with the two PSI compartments, six distinct SAS were resolved in ΔPSII. To estimate the equilibria, spectral area constraints were applied (van Stokkum et al., 2023b). Moreover, the initial excitation conditions (excitation vectors) were set for each experimental condition based on plausible absorbance cross sections of chromophore groups.

All estimated SAS agree well with the respective emission spectra in the literature (Chukhutsina et al., 2013; van Stokkum et al., 2018), which is a primary criterion to evaluate that the pigment groups are correctly identified in the model. The most important finding is the direct EET from the terminal emitter of the PB (APC680) to the bulk of PSI at a rate of 42 ns^-1^ at room temperature. Notably, the rod-to-core and intracore dynamics within the PB serve as a rate-limiting step in the energy funnel. Intriguingly, in the absence of PSII, the energy is transferred to PSI with a rate comparable to the observed rate of EET from PB to PSII in PSI-deficient mutants (Acuña et al., 2018), where a rate constant of 50 ns^-1^ was estimated.

From the simultaneous target analysis of the four different excitation experiments, we also estimated the fractions of the different megacomplexes (**Table 1**). Moreover, on comparing the time-dependent concentration profile obtained from the target model of the isolated PB complex and ΔPSII cells, we could clearly see a faster quenching of the core pigments APC660 and APC680 compared to the PB, cf. the dashed vs. the solid red and black lines in **Figure 6C**. This is direct evidence for the EET to PSI.

**Figure 7:**
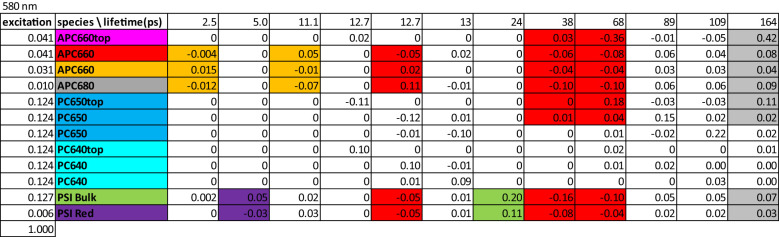
Amplitude matrix of the PB-PSI complex at RT with 580 nm excitation. Color code of the species and estimated microscopic rates are given in **Figure 5**. Color code of the largest amplitudes indicates equilibration between compartments. Further explanation in the text.

The amplitude matrix (**Figure 7**) describes the contributions of each lifetime to the concentrations of the species with *λ_exc_* 580 nm. The fully equilibrated complex decays with a 164 ps lifetime (right-hand column). This explains the longest lifetimes observed in the global analysis (**Figure 3**). The purple and green shading indicate the equilibration in 5 ps and the trapping in 24 ps in the directly excited PSI, in agreement with the observed PSI lifetimes in the global analysis (**Figure 3A**). The orange shading indicates the equilibration in the bottom cylinders with 2.5 and 11.1 ps. The red shading (13, 38 and 68 ps) indicates the equilibration between the bottom cylinders of the core and PSI. The rods connected to the red APC 660 compartment decay with 89 ps, whereas the rods connected to the orange APC 660 compartment decay with 109 ps. Intra-rod equilibration is in ≈13 ps. All these lifetimes mix up in the equilibration time scales observed in the global analysis (**Figure 3**).

### Outlook

Recently, we demonstrated that the EET from the terminal emitter of the PB to the bulk of PSI at 77K (van Stokkum et al., 2023a) can be described with the same rate of 42 ns^-1^ that is found here at room temperature. This firmly establishes the direct EET from PB to PSI. Now that we have demonstrated the energy transfer pathways in the two mutants which lack either PSI (Acuña et al., 2018) or PSII (this work), the next challenges are to establish those pathways in WT cells, and in mutants lacking particular APC680 pigments (Jallet et al., 2012).

## Data availability statement

The original contributions presented in the study are included in the article/**Supplementary Material**. Further inquiries can be directed to the corresponding author.

## Author contributions

AB: Conceptualization, Data curation, Formal analysis, Investigation, Writing – original draft. PA: Conceptualization, Formal Analysis, Investigation, Supervision, Writing – original draft, Writing – review & editing. PL: Conceptualization, Funding acquisition, Investigation, Resources, Supervision, Writing – original draft, Writing – review & editing. IvS: Conceptualization, Formal analysis, Investigation, Methodology, Software, Supervision, Writing – original draft, Writing – review & editing.
