# Supplementary material for: Energy transfer from phycobilisomes to photosystem I at room temperature
Source: Front Plant Sci. 2024 Jan 8;14:1300532. doi: 10.3389/fpls.2023.1300532 (PMC10800844; doi:10.3389/fpls.2023.1300532)
Supplement: Supplementary file 1 [file DataSheet_1.pdf]

Supplementary Material to

Energy transfer from phycobilisomes to photosystem I at room temperature

Avratanu Biswas[1,2,3], Parveen Akhtar[2], Petar H. Lambrev[2] and Ivo H.M. van Stokkum[1,*]

1 Department of Physics and Astronomy and LaserLaB, Faculty of Science, Vrije Universiteit Amsterdam, De Boelelaan 1081, 1081 HV, Amsterdam, The Netherlands

2 HUN-REN Biological Research Centre, Temesvári krt. 62, Szeged 6726, Hungary;
3 Doctoral School of Biology, University of Szeged, Közép Fasor 52, Szeged 6726, Hungary.

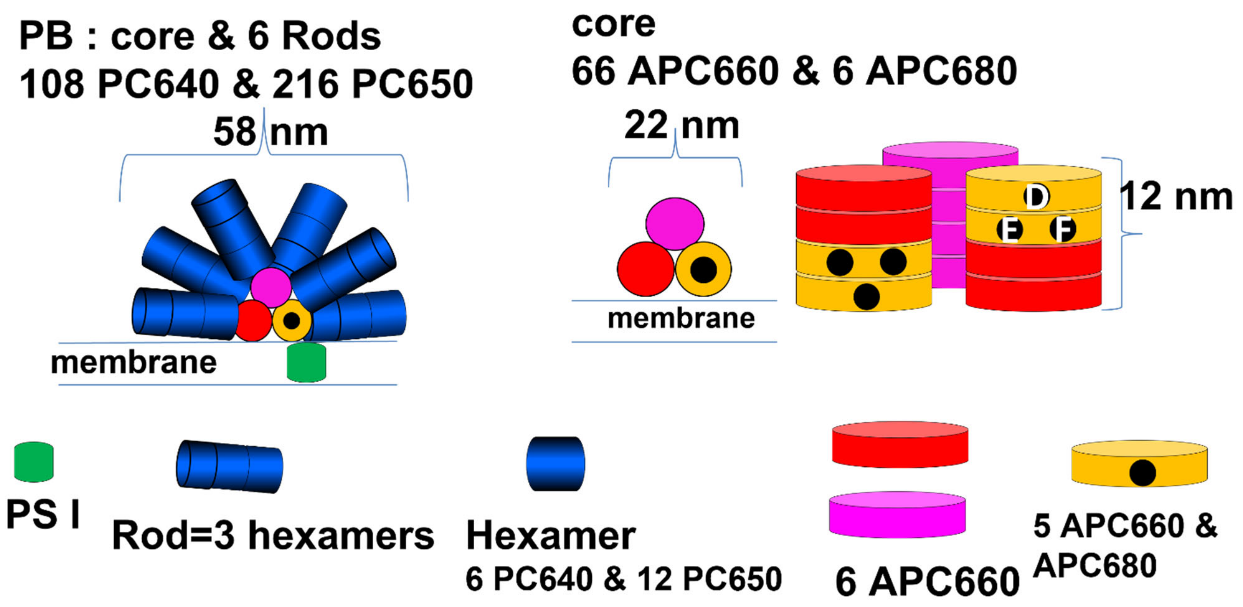

Figure S 1. Cartoon in side-view of a PB-PSI complex in the *Synechocystis* ΔPSII mutant with stoichiometry. *Key:* blue, rods consisting of three hexamers; top and basal core cylinders respectively in magenta, red and orange; green, PS I trimer. Color code as in Figure 5. The letters D,E,F indicate the three different APC680 pigments. The approximate length for each subunit is based on (Arteni et al., 2009). Figure adapted from (Acuña et al., 2018).

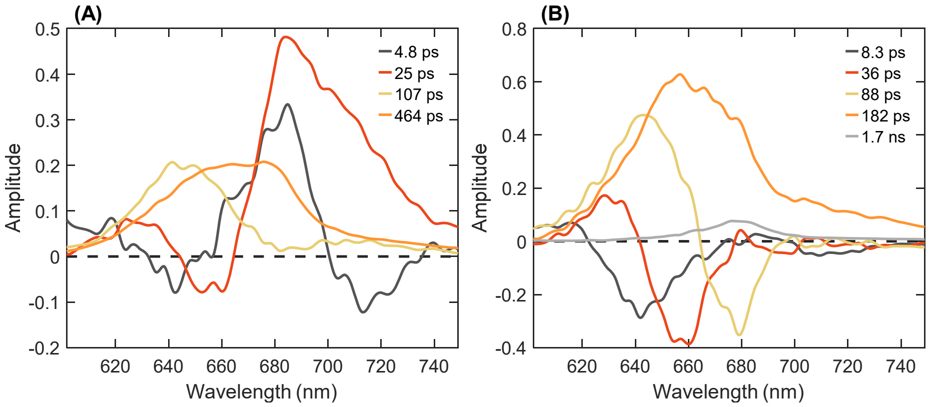

Figure S 2. DAS of *Synechocystis* WT cells obtained from global analysis of time-resolved fluorescence decays measured at RT. (A) Excitation wavelength 400 nm; (B) Excitation wavelength 580 nm.

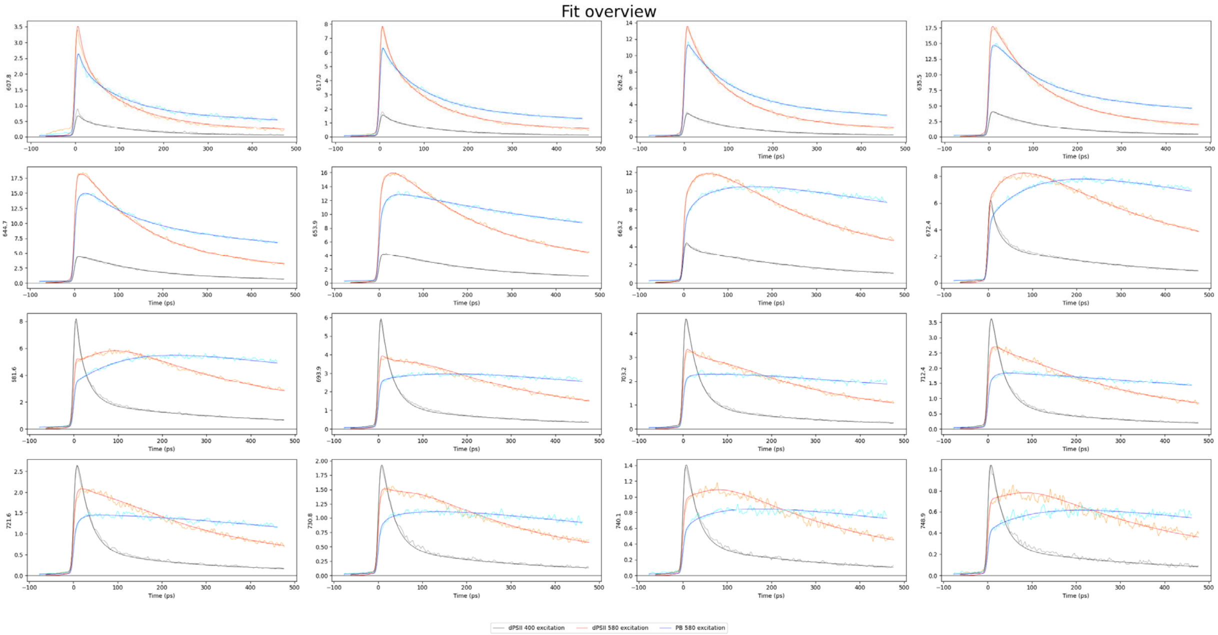

Figure S 3. Selected time traces of the emission of the PB and the ΔPSII mutant cells at 16 wavelengths (indicated in the ordinate label of the panels) after 580 or 400 nm excitation. Key: 400 ΔPSII (grey), 580 ΔPSII (orange), 580 PB (cyan). Black, red, and blue lines indicate the simultaneous target analysis fit. Overall rms error of the fit was 0.061.

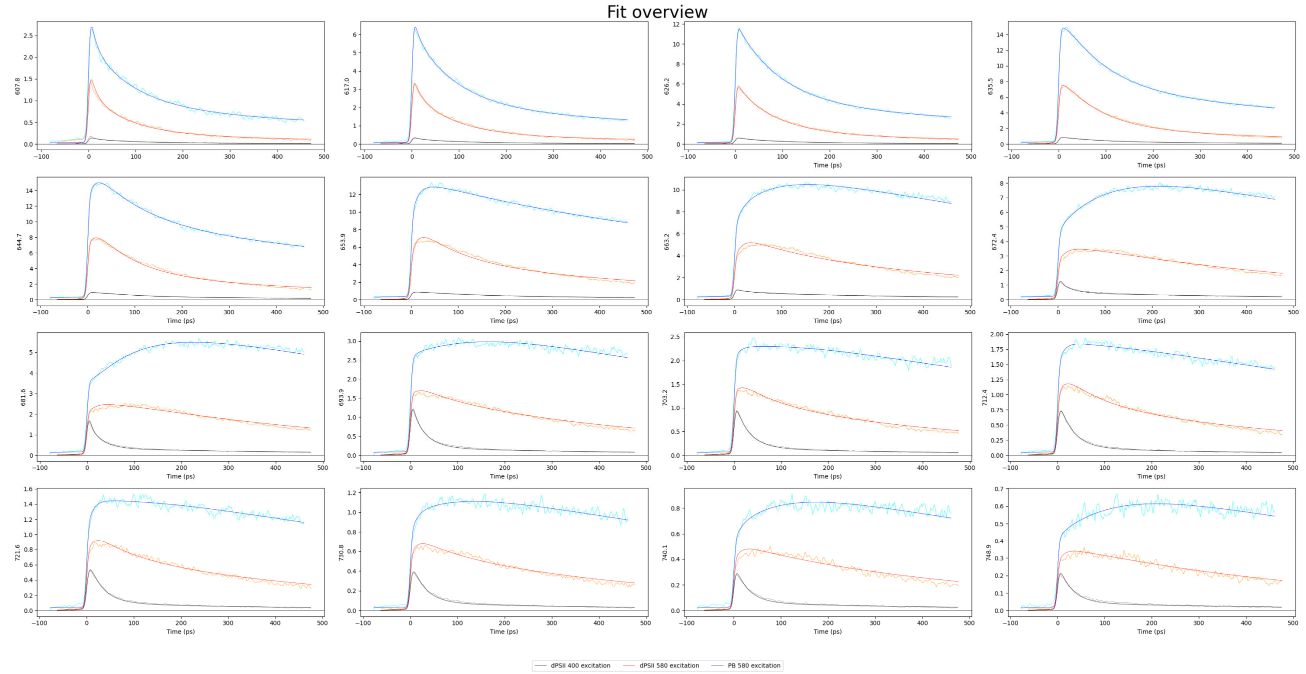

Figure S 4. Selected time traces of the emission of the PB and the ΔPSII mutant cells at 16 wavelengths (indicated in the ordinate label of the panels) after 580 or 400 nm excitation. Key: 400 ΔPSII (grey), 580 ΔPSII (orange), 580 PB (cyan). Black, red, and blue lines indicate the **alternative** target analysis fit with EET to PSI from the PC650 instead of the APC680. Overall rms error of the fit was 0.102.

References

ACUÑA, A. M., VAN ALPHEN, P., VAN GRONDELLE, R. & VAN STOKKUM, I. H. M. 2018. The phycobilisome terminal emitter transfers its energy with a rate of (20 ps)–1 to photosystem II. *Photosynthetica,* 56**,** 265-274.

ARTENI, A. A., AJLANI, G. & BOEKEMA, E. J. 2009. Structural organisation of phycobilisomes from Synechocystis sp strain PCC6803 and their interaction with the membrane. *Biochimica Et Biophysica Acta-Bioenergetics,* 1787**,** 272-279.
